# 

**Published:** 1872-03-15

**Authors:** 


Money Receipts to March 1st, 1872.—Drs. Peck and Moore, $3.00; Dr. F. N. Foote, $3.00; H. Wardner, $6.00; J. O. Harriott, $3.00; C. H. Harvey, $3.00; N. Shilling, $3.00; S. S. Strayer, $3.00; D. J. McMillan, $3.00; W. H. Byford, $6.00; R. C. Hamill, $3.00; Thos. Bevan, $6.00; S. J. Jones, $6.00; A. Fisher, $3.00; B. Wilson, $3.00; T. D. Palmer, $9.00; Thomas L. Jacker, $9.00; Joseph Tefft, $5.00; J. A. Parmenter, $3.00; W. P. Welch, $3.00; E. A. Shafer, $2.00; M. O. Heydock, $3.00; C. S. Ford, $6.00; S. Marks, $3.00; D. Norcom, $6.00; I. N. Lilly, $3.00; S. D. Humphrey, $3.00; H. W. Kendall, $6.00; R. Ludlam, $3.00; C. Goodbroke, $3.00; C. C. Reichard, $3.00.

